# Decline in Child Marriage and Changes in Its Effect on Reproductive Outcomes in Bangladesh

**DOI:** 10.3329/jhpn.v30i3.12296

**Published:** 2012-09

**Authors:** S.M. Mostafa Kamal

**Affiliations:** Department of Mathematics, Islamic University, Kushtia 7003, Bangladesh

**Keywords:** Age at first birth, Child marriage, Child mortality, Induced abortion, Reproductive outcomes, Unintended pregnancy, Bangladesh

## Abstract

This paper explores the decline in child marriage and changes in its effect on reproductive outcomes of Bangladeshi women, using the 2007 Bangladesh Demographic and Health Survey data. Chi-square tests, negative binomial Poisson regression and binary logistic regression were performed in analyzing the data. Overall, 82% of women aged 20-49 years were married-off before 18 years of age, and 63% of the marriages took place before 16 years of age. The incidence of child marriage was significantly less among the young women aged 20-24 years compared to their older counterparts. Among others, women's education appeared as the most significant single determinant of child marriage as well as decline in child marriage. Findings revealed that, after being adjusted for sociodemographic factors, child marriage compared to adult marriage appeared to be significantly associated with lower age at first birth (OR=0.81, 95% CI=76-0.86), higher fertility (IRR=1.45, 95% WCI=1.35-1.55), increased risk of child mortality (IRR=1.64, 95% WCI=1.44-1.87), decreased risk of contraceptive-use before any childbirths (OR=0.56, 95% CI=0.50-0.63), higher risk of giving three or more childbirth (OR=3.94, 95% CI=3.38-4.58), elevated risk of unplanned pregnancies (OR=1.21, 95% CI=1.02-1.45), increased risk of pregnancy termination (OR=1.16, 95% CI=1.00-1.34), and higher risk of the use of any current contraceptive method (OR=1.20, 95% CI=1.06-1.35). Increased enforcement of existing policies is crucial for the prevention of child marriage. Special programmes should be undertaken to keep girls in school for longer period to raise the age of females at first marriage in Bangladesh and thereby reduce the adverse reproductive outcomes.

## INTRODUCTION

Marriage marks the onset of a socially-acceptable institution in a society where offspring procreation occurs within marital union and illegitimate childbirths are rare. It is an important and memorable episode of life for men as well as women. Age at first marriage is more important for young people as it marks the beginning of transition from adolescence to adulthood. Timing of the first marriage or union is an important dimension of women's reproductive behaviour, with far-reaching consequences, particularly for their reproductive health and social status (1). Thus, age at marriage is worthy of attention due to close linkage between marriage and various reproductive and social outcomes.

Child marriage (before the age of 18 years) and marriage at very young age (before the age of 16 years) are a reality for many women (2). The practice of marrying girls at young age is very common in sub-Saharan Africa and South Asia. Globally, 36% of women aged 20-24 years were married or in union before they reached the age of 18 years whereas the incidence was 65% in Bangladesh—the second highest rate after Niger where the prevalence of child marriage or union was 77% in around 2006 (3). A recent estimate reveals that Bangladesh ranked the third in the prevalence of child marriage (69%) in the world (4). Poverty, protection of girls, family honour, and the provision of stability during unstable social periods are predicted to be significant factors in determining the causes of child marriage (2).

Marriage at a very young age has grave social, psychological and health consequences for both young women and their children. Child marriage is not only recognized as a human rights violation, it is also a barrier to individual and social development. Considerable evidence shows that the negative consequences of child marriage are numerous, especially harmful for girls, their children, and their communities (4). Previous research pointed out a variety of social, familial, health and financial outcomes which are strongly correlated with early teenage marriage and low education. For instance, the women who marry in their teens tend to have more children and have childbirth earlier than their adult peers. The negative outcomes associated with early marriage and dropping out of high school have the potential to affect not only the individual decision-making but also children and the rest of the society (4).

Furthermore, girls already in school are often forced to terminate their education when they marry early (5). Limited mobility, household responsibilities, pregnancy and rearing children, and other social restrictions for married girls prevent them from taking advantage of education or work opportunities (6). Opportunities for young mothers to continue their education or to work are often limited because they have little access to resources and are responsible for childrearing and household tasks (4). The women married at early age are more likely than those who are married-off as adult to have early, frequent and unplanned pregnancies, typically from lack of contraceptive-use (7). The children of teenage mothers experience serious health consequences as well. A child born to a teenage mother is twice as likely to die before his/her first birth day than the child of a woman in her twenties (4). If they survive, these infants tend to have higher rates of low birthweight, premature birth, and infant mortality than those born to older mothers (5).

In Bangladesh, as in many other countries of South Asia, marriage is nearly universal and women marry at a very young age (8). Bangladesh has a long tradition of early marriage and early motherhood. The Muslim Family Ordinance 1961 (amended in 1981) of the Bangladesh Government has set the minimum age at first marriage for males and females at 21 years and 18 years respectively. However, the Ordinance is hardly followed. Studies on age of females at marriage, particularly child marriage and its social and health consequences among women of Bangladesh, are limited. Additionally, research conducted so far on age at marriage in Bangladesh has focused mainly on examining the customs and factors affecting age of females at first marriage (9-12). A little attention has been paid to reproductive outcomes associated with teenage marriage in Bangladesh. This paper aims to investigate the prevalence of and decline in child marriage and its effect on various reproductive outcomes among Bangladeshi women, using the nationally-representative 2007 Bangladesh Demographic and Health Survey (BDHS) data.

We hypothesize that education has a significantly negative effect on child marriage. We also hypothesize that the effect of education is largely independent of current age of women, type of place of residence, religion, region of residence, and wealth index. Furthermore, we hypothesize that women of younger generations are less likely to be married as a child compared to those of the older generations due to increased level of education over time. In addition to these, we hypothesize that child marriage is significantly associated with lower age at first birth, higher fertility and child mortality, lower use of any contraceptive method before any childbirth, increased risk of unintended pregnancy, and pregnancy termination.

## MATERIALS AND METHODS

### Data

The study used data from the 2007 BDHS—the nationally-representative survey that gathered information from 10,996 ever-married women aged 15-49 years and 3,771 men aged 15-54 years from 10,400 households. The survey used a multistage cluster sample based on the 2001 Bangladesh Census. The survey obtained detailed information on fertility levels, marriage, fertility preferences, awareness, and use of family-planning methods, breastfeeding practices, nutritional status of women and young children, childhood mortality, maternal and child health, knowledge and attitudes regarding HIV/AIDS, etc. The survey was conducted under the authority of the National Institute for Population Research and Training (NIPORT) of the Ministry of Health and Family Welfare. The details of the survey have been described in the survey report (13).

### Sample-size

The study dealt with the weighted sample of 9,572 ever-married women aged 20-49 years. The sample was made weighted by the corresponding weighting factor provided for each of the sample women in the SPSS raw data. The ever-married female adolescents aged 15-19 years were excluded from analyses as almost half of the female adolescents remained unmarried during the survey and their inclusion may bias the findings.

### Dependent variables

For deepening our understanding of the extent to which child marriage has declined over time and of the factors causing child marriage and marriage at very young age, the first part of this study dealt with two dependent variables: marriage at very young age (marriage before 16 years of age) and child marriage (marriage before 18 years of age). In the second part, we assessed the effect of child marriage on the adverse outcomes of various reproductive behaviours of women. In this part, the demographic and fertility-related dependent variables included for analyses are: age at first birth, number of children ever born, number of children who died, use of family-planning (FP) method before any childbirth, childbirth within one year of marriage, three or more childbirths, whether the most recent pregnancy was intended or unintended, ever had terminated pregnancy, and current use of any FP method.

### Independent variables

To assess the net effect of marriage before 16 years of age and marriage before 18 years of age, six vital factors were included as the independent variables: current age of the women, women's education, current place of residence, geographical region, religion, and wealth index. Year of marriage was considered an independent variable in bivariate analysis but was not included in multivariate analyses to avoid multicollinearity with current age of women. Along with these variables, age at first marriage was included as an independent variable to examine the effect of child marriage on the reproductive behaviours of women. The definitions and coding of the dependent and the independent variables considered for analyses are presented in Table 1.

### Statistical analyses

Both bivariate and multivariate statistical analyses were employed in this study. The associations of very young marriage and child marriage with sociodemographic characteristics were assessed by chi-square tests, with significance for all analyses set at p<0·05. This was followed by logistic regression to assess the net effect of the covariates on the outcome variables. Negative binomial Poisson regression was employed to estimate the incidence of relative risk (IRR) for child marriage on fertility outcomes (for count data), such as age at first birth, number of children ever born, and number of children who died. Besides, a series of binary logistic regression models were constructed to assess the net effect of child marriage on the reproductive behaviours of women, after confounding with other selected independent variables. The results of the negative binomial Poisson regression analyses are presented by regression coefficients, and IRR with 95% Wald confidence interval (WCI) and those for logistic regression analyses are presented by regression coefficients and odds ratios (OR) with 95% confidence interval (CI). The statistical analyses applied in this study were performed by SPSS (version 17).

## RESULTS

### Background profile of the respondents

Table 2 shows the mean and frequency distribution of the women by their background characteristics. The mean age of the women was 32.4±8.5 years, and the mean age at first marriage was 15.5±3.0 years. Of the respondents, almost 63.0% got married at very young age, and almost 82.0% were married-off as child. When age at first marriage was broken down into four categories, it showed that 32.2% were married-off at age below 14 years; 30.5% got married at 14-15 years; 19.2% were married-off at 16-17 years of age, and 18.1% marriage took place at 18 years of age or above. Of the women, more than one-third had no formal education, over one-quarter had secondary education, and a slightly over 6.0% had higher education. Over three-quarters of the women lived in rural areas. More women were from Dhaka division, followed by Rajshahi, Chittagong, Khulna, Sylhet and Barisal division. Most women were Muslim. In terms of wealth index, a slightly less than two-fifths were from poor households, and a slightly over two-fifths were from rich households.

### Differentials of marriage at very young age and child marriage

Table 3 shows the differentials of marriage at very young age and child marriage by background characteristics of women. All of the independent variables included for analyses showed highly (p<0.001) significant association with the outcome variables. Both age cohort and marriage cohort of women revealed that child marriage as well as marriage at very young age has declined over time. For instance, the incidence of child marriage was 90.9% among women aged 45-49 years, which consistently decreased to 77.2% among those aged 20-24 years. In addition, the prevalence of marriage before 16 years of age for women aged 45-49 years was 78.5%, which decreased to 53.2% for women aged 20-24 years. The marriage cohort of women showed that, although very young marriage was nearly universal for women married in the 1967-1974 period, the prevalence declined to 26% for marriage during 2000-2007. It is notable that child marriage was universal for women who were married-off during 1967-1974; it decreased to 54.4% among women who got married during 2000-2007. Women's education apparently showed negative association with child marriage. The prevalence of marriage for both categories: before 16 years of age as well as before 18 years of age was significantly higher in rural than in urban areas. The incidence of child marriage and marriage at very young age was more prevalent in Rajshahi division, followed by Khulna, Barisal, Dhaka, Chittagong and Sylhet division. Child marriage and marriage at very young age were more prevalent among the Muslim than the non-Muslim women. Wealth index showed negative association with child marriage.

**Table. 1.** Operational definitions of dependent and independent variables and their measurements

Variable	Description	Measurement scale
Dependent variables:		
Marriage at very young age	Whether the women were married before 16 years of age	Dichotomous 0=No and 1=Yes
Child marriage	Whether the women were married before 18 years of age	Dichotomous 0=No and 1=Yes
Age at first birth	The age of women at the time of first childbirth	Continuous (count data)
Children ever born	Number of children born to an ever-married woman	Continuous (count data)
Children died	Number of children who died to an ever-married woman	Continuous (count data)
Used FP before any childbirth	Whether a woman used contraceptive method before her first childbirth	Dichotomous 0=No and 1=Yes
Childbirth within one year of marriage	Whether a woman gave any livebirth within one year of her first marriage	Dichotomous 0=No and 1=Yes
Three or more children	Whether a woman gave three livebirths during her reproductive period	Dichotomous 0=No and 1=Yes
Most recent pregnancy was unwanted or mistimed	Whether current pregnancy and the most recent birth, hereafter, and most recent pregnancy were unintended	Dichotomous 0=No and 1=Yes
Ever had terminated pregnancy	Whether a woman ever terminated pregnancy in her reproductive period	Dichotomous 0=No and 1=Yes
Currently using any contraceptive method	Respondent who were currently using any contraceptive method	Dichotomous 0=No and 1=Yes
Independent variables:		
Age at marriage	Age at first marriage of women	Ordinal 1=Child marriage; and 2=Adult marriage
Current age of women	Respondent's current age at the time of survey	(i) Continuous and (ii) Ordinal 1=20-<25; 2=25-<30 3=30-<35; 4=35-<40 5=40-<45 and 6=45-49
Year of marriage	In which year a woman was married-off	Ordinal 1=1967-1974; 2=1975-1979 3=1980-1984; 4=1985-1989 5=1990-1994; 6=1995-1999 and 7=2000-2007
Women's education	Educational level of women	Ordinal 0=No formal education 1=Primary 2=Secondary or above
Current residence	Current place of residence	Ordinal 1=Urban and 2=Rural
Region	Place of region	Ordinal 1=Barisal; 2=Chittagong 3=Dhaka; 4=Khulna 5=Rajshahi; and 6=Sylhet
Religion	Religious affiliation	Ordinal 1=Muslim and 2=Non-Muslim
Wealth index	Luxurious materials available in household	Ordinal 1=Poorest; 2=Poorer 3=Middle; 4=Richer 5=Richest

**Table. 2.** Mean with standard deviation (SD) and weighted percentage distribution of women by their background characteristics

Characteristics	Sample women		Mean±SD
	No.	%	
Current age (years)			32.4±8.5
20-<25	2,175	22.7	
25-<30	1,931	20.2	
30-<35	1,660	17.3	
35-<40	1,564	16.3	
40-<45	1,213	12.7	
45-49	1,030	10.8	
Year of marriage			
1967-1974	701	7.3	
1975-1979	938	9.8	
1980-1984	1,267	13.2	
1985-1989	1,529	16.0	
1990-1994	1,659	17.3	
1995-1999	1,816	19.0	
2000-2007	1,661	17.4	
Age at first marriage (years)			15.3±2.8
<14	3,083	32.2	
14-<16	2,920	30.5	
16-<18	1,834	19.2	
18+	1,736	18.1	
Women's education			
No education	3,603	37.3	
Primary	2,856	29.8	
Secondary	2,502	26.1	
Higher	609	6.4	
Current residence			
Urban	2,230	23.3	
Rural	7,342	76.7	
Region			
Barisal	577	6.0	
Chittagong	1,766	18.4	
Dhaka	3,003	31.4	
Khulna	1,219	12.7	
Rajshahi	2,380	24.9	
Sylhet	627	6.6	
Religion			
Islam	8,670	90.6	
Others	902	9.4	
Wealth index			
Poorest	1,876	19.6	
Poorer	1,835	19.2	
Middle	1,862	19.5	
Richer	1,955	20.4	
Richest	2,044	21.4	
Total	9,572	100.0	

**Table. 3.** Prevalence of marriage at age <16 years and at age <18 years among Bangladeshi women by background characteristics

Characteristics	AAM <16 years		Chi-square		AAM <18 years		Chi-square
	No	Yes			No	Yes	
Current age (years)			255.3***				125.1***
20-<25	46.8	53.2			22.8	77.2	
25-<30	42.4	57.6			21.2	78.8	
30-<35	35.6	64.4			18.4	81.6	
35-<40	36.4	63.6			17.7	82.3	
40-<45	28.9	71.1			12.7	87.3	
45-49	21.5	78.5			9.1	90.9	
Year of marriage			1571.7***				1218.6***
1967-1974	2.1	97.9			0.0	100.0	
1975-1979	19.3	80.7			4.5	95.5	
1980-1984	26.2	73.8			9.8	90.2	
1985-1989	31.7	68.3			12.1	87.9	
1990-1994	34.1	65.9			16.2	83.8	
1995-1999	41.9	58.1			19.7	80.3	
2000-2007	74.0	26.0			45.6	54.4	
Women's education			1284.6***				1550.7***
No education	24.1	75.9			9.0	91.0	
Primary	29.4	70.6			12.1	87.9	
Secondary	52.9	47.1			25.1	74.9	
Higher	88.4	11.6			72.1	27.9	
Current residence			155.4***				211.1***
Urban	48.5	51.5			28.5	71.5	
Rural	33.9	66.1			15.0	85.0	
Region			243.4***				177.1***
Barisal	35.8	64.2			14.9	85.1	
Chittagong	47.0	53.0			24.6	75.4	
Dhaka	35.8	64.2			18.6	81.4	
Khulna	31.3	68.7			13.7	86.3	
Rajshahi	30.3	69.7			12.6	87.4	
Sylhet	56.7	43.3			29.9	70.1	
Religion			99.2***				106.2***
Islam	35.7	64.3			16.8	83.2	
Others	52.6	47.4			30.8	69.2	
Wealth index			506.8***				626.6***
Poorest	22.9	77.1			8.3	91.7	
Poorer	30.7	69.3			12.4	87.6	
Middle	34.4	65.6			14.1	85.9	
Richer	41.0	59.0			20.4	79.6	
Richest	55.5	44.5			33.8	66.2	
Total	37.3	62.7			18.1	81.9	

Level of significance

***p<0.001;

AAM=Age at marriage

**Table. 4.** Mean and percentage distribution of various indicators of reproductive behaviours by background characteristics of women in Bangladesh

Reproductive behaviour	Mean or percentage of women (N)		
	Overall	Adult marriage	Child marriage
Age at first birth (mean)	17.8 (SD±3.2)	21.8 (SD±2.8)	17.0 (SD±2.6)
Children ever born (mean)	3.1 (SD±2.0)	2.0 (SD±1.6)	3.3 (SD±1.6)
Children died (mean)	0.4 (SD±0.8)	0.2 (SD±0.5)	0.5 (SD±0.8)
Used FP before any childbirth				
No	65.9 (6302)	49.1 (851)	69.6 (5451)
Yes	34.1 (3267)	50.9 (884)	30.4 (2383)
Childbirth within one year of marriage				
No	78.1 (7092)	71.9 (1062)	79.3 (6930)
Yes	21.9 (1985)	28.1 (415)	20.7 (1570)
Three or more children			
No	45.3 (4337)	73.3 (1272)	39.1 (45.3)
Yes	54.7 (5235)	26.6 (464)	60.9 (54.7)
Most recent pregnancy was unwanted or mistimed				
No	66.4 (2873)	71.3 (709)	64.9 (2164)
Yes	33.6 (1457)	28.7 (285)	35.1 (1172)
Ever had terminated pregnancy				
No	79.0 (7549)	82.4 (1427)	78.2 (6122)
Yes	21.0 (2012)	17.4 (306)	21.8 (1706)
Currently using any FP			
No	46.1 (4414)	47.8 (831)	45.7 (3583)
Yes	53.9 (5158)	52.2 (905)	54.3 (4253)

SD=Standard deviation

### Comparison of different reproductive outcomes between adult and child marriage

Table 4 shows the comparison of different reproductive outcomes between women married-off as child and as adult. The mean age at first birth for women married as child and as adult was 17.0 years and 21.8 years respectively. The mean number of children ever born to an ever-married woman was 3.1. The corresponding figures for women married as child and as adult were 3.3 and 2.0 respectively. Furthermore, the mean number of children who died in case of ever-married women was 0.4. The difference in mean child mortality between women married at adulthood and childhood was 0.2, with the prevalence being higher among those who were married at childhood. The non-use of any contraceptive method before any childbirth was almost 70% in the women who got married at their childhood and 49% among those who were married-off as adults. A slightly over one-fifth of the women married at childhood and 28% of the women married at adulthood reported to have had a childbirth in the first year of marriage. At least three-fifths of the women who were married-off at their childhood had three or more children opposed to 27% who got married as adults. Overall, 35% women married at childhood reported that their most recent pregnancies were unintended whereas this incidence was 29% in the women married as adults. The prevalence of pregnancy termination rate was 4% higher among women married at childhood than their adult counterparts. The current contraceptive prevalence rate was 2% higher among women married at childhood than their adult peers.

### Results of regression analyses

Table 5 shows the multivariate logistic regression analyses for marriage at very young age as well as the child marriage. All independent variables included in the analyses showed to have significant relationship with outcome variables. For both outcome variables, we introduced interaction terms for current age and education level of women to examine the effect of education on child marriage and to assess whether there was any pattern of decline in child marriage over time through education.

Current age of women, after being adjusted for other covariates, did not show any apparent pattern of decline in marriage at very young age and child marriage. For instance, compared to women aged 20-24 years, the women aged 45-49 years were two times likely to be married-off at very young age. However, the difference in the likelihood of being married at very young age between women aged 20-24 to 40-44 years was not found to be significant. Women aged 45-49 years compared to the young women aged 20-24 years were almost two times likely and those aged 25-29 years were also likely to be married-off as child by the factor 1.64. The difference in the likelihood of being married-off as child between women aged 20-24 years and 30-34 to 40-44 years was not found to be significant. Overall, the child marriage has significantly declined in women of younger generation than those aged 45-49 years.

Women's education showed a significantly negative relationship with the timing of marriage when other sociodemographic covariates were kept constant. For instance, women with primary, secondary and higher education, compared to those with no formal education, were respectively 24%, 72%, and 94% less likely to be married at very young age. Almost a similar pattern of decline in child marriage was observed for this variable. Compared to the urban women, the rural women were more likely to be married-off at very young age and be married as child by factor 1.21 and 1.35 respectively. The women of Chittagong and Sylhet division were less likely and those of Khulna and Rajshahi division were more likely to be married at very young age compared to those of Barisal division. Besides, the women of Chittagong, Sylhet and Dhaka division were less likely to get married before 18 years of age compared to women of Barisal division.

The likelihood of being married-off at very young age and child marriage was 52% and 59% less among the non-Muslim women than their Muslim peers. The wealth quintiles showed consistently a negative association with marriage at very young age; child marriage decreased significantly among women from richer and the richest families. The current age and women's education produced 13 interaction terms, in which two and four terms appeared to be significant for marriage at very young age and child marriage respectively. The significant interaction terms as shown in Table 5 suggest that women's higher education is a powerful predictor that negatively influences the child marriage.

Table 6 demonstrates the unadjusted and adjusted effect of child marriage on various reproductive behaviours of women. The inclusion of socioeconomic factors substantially attenuated the effect of the timing of first marriage on the reproductive outcomes. The adjusted estimated coefficients of child marriage obtained through negative binomial Poisson regression analyses for age at first birth appeared as negative (β=-0.22) whereas the estimated coefficients for the number of children ever born (β=0.37) and number of children who died (β=0.50) appeared as positive and statistically significant. The exponentiated effect parameters adjusted for sociodemographic factors suggest that, for increment of each year, age at first birth decreased by 19% (IRR=0.81, WCI=0.76-0.86) among women married as child compared to their adult married peers. The cumulative fertility and cumulative child mortality increased by 45% (IRR=1.45, WCI=1.35-1.55) and 64% (IRR=1.64, WCI=1.44-1.87) respectively among the women married as child than their adult married counterparts.

The multivariate logistic regression analysis, adjusted for relevant socioeconomic and demographic factors, showed that the women married as child were significantly (p<0.001) less likely to report using any contraceptive method (OR=0.56, CI=0.50-0.63) before any childbirth than those who were married-off as adult. Childbirth within one year of marriage was significantly lesser by the factor 0.67 (OR=0.67, 95% CI=0.58-0.77) among the women who got married as child than women who were married-off as adult. The women married as child were significantly more likely to have three or more children (OR=3.94, 95% CI=3.38-4.58), increased risk of unintended recent pregnancy (OR=1.21 95% CI=1.02-1.45), boosted risk of pregnancy termination (OR=1.16, 95% CI=1.00-1.34), and elevated risk of current contraceptive-use (OR=1.20, 95% CI=1.06-1.35) compared to women who got married as adult.

**Table. 5.** Multivariate logistic regression analyses showing the effect of marriage at age <16 years and at age <18 years among Bangladeshi women by background characteristics

Characteristics	Marriage at age <16 years				Marriage at age <18 years		
	β	OR	95% CI		β	OR	95% CI
Current age (years)							
20-<25	-	1.00	-		-	1.00	-
25-<30	0.04	1.05	0.76-1.43		0.49	1.64*	1.02-2.64
30-<35	0.15	1.16	0.86-1.58		0.11	1.12	0.72-1.73
35-<40	-0.04	0.96	0.71-1.29		0.05	1.06	0.69-1.61
40-<45	0.05	1.05	0.78-1.43		-0.10	0.90	0.59-1.38
45-49	0.69	1.99***	1.43-2.77		0.65	1.91**	1.18-3.08
Women's education							
No education	-	1.00	-		-	1.00	-
Primary	-0.27	0.76*	0.57-1.03		-0.30	0.74	0.49-1.12
Secondary	-0.1.28	0.28***	0.21-0.37		-1.04	0.35***	0.24-0.52
Higher	-2.79	0.06***	-0.4-0.10		-2.86	0.06***	0.04-0.09
Current residence							
Urban	-	1.00	-		-	1.00	-
Rural	0.19	1.21**	1.07-1.38		0.30	1.35***	1.15-1.57
Region							
Barisal	-	1.00	-		-	1.00	-
Chittagong	-0.44	0.64***	0.52-0.80		-0.68	0.51***	0.38-0.68
Dhaka	0.04	1.04	0.84-1.28		-0.27	0.76*	0.58-1.01
Khulna	0.28	1.32**	1.05-1.66		0.15	1.16	0.85-1.60
Rajshahi	0.24	1.27**	1.03-1.57		0.17	1.18	0.89-1.58
Sylhet	-1.14	0.32***	0.25-0.41		-1.31	0.27***	0.20-0.37
Religion							
Islam	-	1.00	-		-	1.00	-
Others	-0.74	0.48***	0.41-0.57		-0.90	0.41***	0.34-0.49
Wealth							
Poorest	-	1.00	-		-	1.00	-
Poorer	-0.21	0.82**	0.70-0.95		-0.17	0.85	0.68-1.06
Middle	-0.24	0.78***	0.67-0.92		-0.17	0.84	0.68-1.06
Richer	-0.32	0.72***	0.62-0.85		-0.34	0.71***	0.57-1.88
Richest	-0.48	0.62***	0.52-0.74		-0.45	0.64***	0.50-0.81
Interaction terms							
Age-group*against women's education						
(25-29)*secondary					-0.53	0.59*	0.34-1.01
(25-29)*higher					-0.90	0.41**	0.21-0.80
(35-39)*primary	0.36	1.43*	0.94-2.19		0.58	1.79*	0.98-3.25
(35-39)*secondary	0.88	2.41***	1.52-3.82		0.97	2.63***	1.41-4.91

Level of significance

***p<0.001;

**p<0.01;and

*p<0.05

**Table. 6.** Regression analysis showing the effect of child marriage on various indicators of reproductive behaviour of women§ of Bangladesh

Reproductive behaviour	Unadjusted IRR/OR with 95% CI				Adjusted IRR/OR with 95% CI		
	β^a^	IRR/OR	95% WCI/CI		β	IRR/OR	95% WCI/CI
Age at first birth‡	-0.25	0.78***	0.74-0.82		-0.22	0.81***	0.76-0.86
Children ever born†	0.50	1.66***	1.56-1.76		0.37	1.45***	1.35-1.55
Children died†	0.85	2.34***	2.07-2.65		0.50	1.64***	1.44-1.87
Used FP before any childbirth†								
No	-	1.00	-		-	1.00	-
Yes	-0.87	0.42***	0.38-0.47		-0.55	0.56***	0.50-0.63
Given childbirth within one year of marriage†								
No	-	1.00	-		-	1.00	-
Yes	-0.41	0.67***	0.59-0.76		-0.42	0.67***	0.58-0.77
Given birth to three or more children†								
No	-	1.00	-		-	1.00	-
Yes	1.45	4.27***	3.80-4.79		1.14	3.94***	3.38-4.58
Most recent pregnancy was unwanted or mistimed†								
No	-	1.00	-		-	1.00	-
Yes	0.30	1.35***	1.15-1.57		0.14	1.21*	1.02-1.45
Ever had terminated pregnancy†								
No	-	1.00	-		-	1.00	-
Yes	0.26	1.30***	1.14-1.49		0.15	1.16*	1.00-1.34
Currently using any FP†								
No	-	1.00	-		-	1.00	-
Yes	0.09	1.09	0.98-1.21		0.13	1.20**	1.06-1.35

^a^The regression coefficients shown for child marriage;

§Indicates that data are missing for some individuals in all subsamples of women;

‡Analyses adjusted for timing of marriage, maternal age, women's education, residence, region, religion, and wealth;

†Analyses adjusted for timing of marriage, current age, women's education, residence, region, religion and wealth; Level of significance

***p<0.001;

**p<0.01; and

*p<0.05

## DISCUSSION

Marriage is an event of great social and cultural importance to women and men in most societies where childbirth is not socially acceptable without marital bond. This study aimed at examining the decline in child marriage in Bangladesh and investigating the effect of child marriage on various reproductive behaviours. The findings of the study reveal that the practice of child marriage is very common in Bangladesh. Overall, the prevalence of child marriage was 82%, and the mean age at first marriage was 15.3 years, indicating that Bangladeshi women were married-off 2.7 years earlier than the legal age at first marriage for females. These statistics suggest that most female marriages still take place before the legal age at first marriage. Findings suggest that current age, women's education, place of residence, socioeconomic status, religion, and regions are important determinants of child marriage in Bangladesh.

The reported prevalence of child marriage reveals that Bangladeshi girls are more vulnerable to child marriage in South Asian context as well as in the world. For instance, a recent study conducted on Indian women reported that 45% of women aged 20-24 years were married-off before 18 years of age (7) whereas our findings showed that 77% of the women aged 20-24 years got married before they reached 18 years of age. Child marriage was more prevalent in the women of western and southern parts of Bangladesh—specifically those regions adjoining to India. Although the age pattern of women showed a decreasing trend in child marriage, it still remains unexpectedly high in Bangladesh. These findings suggest that child marriage is still an important obstacle in programmatic efforts towards women's development and maternal and child health development issues.

Although the bivariate analyses showed apparent pattern of decline in child marriage and marriage at very young age by age cohort of women, the multivariate analyses yielded curvilinear pattern of the likelihood of these two outcome variables. Analyses by year of marriage and age cohort of women reflect that Bangladeshi women are in transition with regard to timing of marriage. For instance, the youngest women were more likely to be married as adult opposed to their older counterparts. This may be partly attributed to the higher educational attainment of younger women compared to their older counterparts as well as socioeconomic transition in the country. This finding is consistent with studies conducted on ethnic tribal women of Bangladesh (14) and elsewhere (15).

Women's education is the most significant single determinant of child marriage in Bangladesh. Typically, higher education of the respondents is likely to be associated with lower probability of early marriage (14). Consistent with earlier studies (14,16,17), the findings of this study reveal that, the higher the educational level the lower the probability of child marriage. It is likely that a woman with higher education has to spend longer time in schooling. In addition, the women who have attained higher education would usually have higher occupational aspiration and would want to have jobs suitable for them rather than getting married earlier. Hence, due to prolonged schooling and desire for career building, the more-educated women are more likely to marry at later ages than others. This implies that girls should be kept in school for a longer period, not only for the purpose of raising age at marriage but also for biological, physical and mental maturity (14).

The patterns of covariates of marital timing among the women under study reflect a society in evolution (14). Place of residence partially explains the socialization process. Besides, religion is an indicator of faith and cultural tradition. These variables would reflect the family values that a woman would have grown up with, and which would influence how she evaluates the costs and benefits between marriage and remaining single (14). The more prevalence and higher likelihood of child marriage among the Muslims and the rural women reflect their traditional cultural norms, beliefs, values, and behaviour. These findings are also consistent with those of earlier studies conducted elsewhere (16,18).

Marriage at very young age and child marriage varied significantly among the administrative regions of the country. It is likely that discernable culture and regional socioeconomic variations might have impact on the timing of marriage. The regional socioeconomic development disparities are bound to affect the timing of marriage (19). Thus, women residing in the more developed regions are more tended to marry later than their counterparts in the less-developed regions as the majority of them would be educated and have more opportunities for career development outside the home.

The present wealth index of the women does not actually reflect their parental socioeconomic status at the time of their marriage. However, the prevailing culture in Bangladesh reveals that parents, guardians as well as the women and men look for grooms and brides of similar socioeconomic status. Thus, the wealth index is an indirect indicator of socioeconomic status of women, their parents as well as their in-laws’ families. Our findings revealed a significantly negative association between wealth index and marriage at very young age. It is likely that the women with a better economic status are likely to be provided by their parents with more alternatives to early marriage, such as higher education and occupational attainment, resulting in lower likelihood of child marriage among them compared to their poor counterparts. This is supported by the interaction terms for age and education. The significant interaction terms suggest that women's higher education than current age of women is a much more important predictor in reducing child marriage.

In societies where marriage is the precursor of socially-acceptable childbirth, childbearing begins soon after marriage. Early marriage is associated with early childbearing in most cases, particularly in the developing world where the main purpose of marriage is to have children (19). The association between early marriage and early motherhood, therefore, remains strong: most women who marry before 18 years of age would begin to have children before they reach 20 years of age (18). In the Bangladesh context, women do hurry to have childbirth as early as possible to prove their fertility. If a woman, particularly in rural areas and lower-educated segment, makes some delay in the initiation of childbearing, she is blamed that she is infertile and is not biologically capable of childbearing. To escape from this blame and to prove her fertility, most women intend to have childbirth soon after their marriage. Thus, it is expected that women married at earlier stages of life should have early age at first birth.

Consistent with many studies (20-22), our findings reveal that age at first marriage is negatively associated with cumulative fertility. Early marriage does not only mark a woman's early entry into a sexual union and the beginning of exposure to childbearing but is also an important gauge of women's status; this is because the older the woman when she marries the greater the likelihood that she has attended school or has been employed and the greater is her chances of having a more equal relationship with her husband (23). The women married at childhood are more likely to be pregnant for a longer duration of conjugal life than women married as adult, resulting in higher fertility among them.

The higher likelihood of child mortality among women married at childhood may be attributed not only to biological and physical maturity but this is also partly attributed to longer duration of marital life. Young maternal age is probably a marker for one or more other maternal risk factor(s) associated with adverse birth outcomes rather than only an indication of incomplete maternal growth (24). The observed association between early teenage pregnancy and adverse birth outcomes simply reflects the deleterious sociodemographic environment. Besides, pregnant teenagers who confront biological immaturities are causally controversial (25). Studies also showed that young maternal age is not only an independent risk factor for adverse birth outcomes, the increased risk is also attributable to other factors that are related to teenage pregnancy, such as ethnicity, low socioeconomic status, and inadequate prenatal care (26-28). However, our finding is quite in good agreement with the previous studies conducted elsewhere (25,29-31).

The lower likelihood of childbearing within one year of marriage among women married as child compared to their adult peers may be partly attributed to biological and physical immaturity among them. Besides, women married at later ages do hurry to have childbirth as they already have passed substantial time for offspring procreation. Furthermore, the higher risk of unplanned pregnancies among the women married as child may be attributed to failure in proper planning of the timing of childbearing and lower use-rate of family planning methods compared to their adult peers. Studies reveal that transition to parenthood at early age is an important and stressful life-event that involves multiple risk factors, such as psychological, marital and economic issues for new mothers (32-34), particularly when they are younger (35-36), resulting in higher likelihood of unplanned pregnancy which often end up with induced abortion. This finding is also consistent with a recent study conducted on Indian women (7). The higher likelihood of any contraceptive-use among women married as child may be due to the fact that they have already achieved completed family-size as they desired and hence want to stop further childbearing through the use of contraceptive methods.

### Limitations

The study has several limitations. First, it dealt with self-reported data that may have error in reporting the sensitive information, like pregnancy termination, although menstrual regulation, which is a type of voluntary pregnancy termination, is legal in Bangladesh whereas induced abortion is still illegal in the country unless it is necessary to save mother's life. It is likely that women often hide the information of induced abortion to escape from social criticism. Second, many variables have been left, such as parental socioeconomic status, working status at the time of marriage, consanguine marriage, etc. These variables were not included in the analyses due to paucity of data, which are important factors in analyzing child marriage. Third, in Bangladesh, as in many other developing countries, there might have error in reporting the respondents’ age, age at marriage, and age at first birth, etc. Many women of reproductive age in Bangladesh prefer hiding their actual age to prove them young. This is due to the fact that registration of age at birth and even age at marriage is not properly followed and maintained. Despite these limitations, the strength of this study is that it dealt with the nationally-representative standard DHS data mostly used from public-health and demographic perspectives.

### Conclusions

The study shows that child marriage is still common in Bangladesh. The pervasiveness of child marriage and its association with higher fertility and poor control of fertility—factors linked to numerous poor maternal and child health outcomes—urges the crucial need for maintaining proper marriage registration and increased family-planning interventions tailored to married adolescents. Poverty and lower education are two crucial factors for early marriage as well as adverse reproductive outcomes. Thus, the findings of the study further suggest that reducing the prevalence of poverty and the need to remain girls in school for a longer period are necessary not only to raise the age at first marriage but also to promote the individual development and their potential contribution to society for overall development. Programmes should be undertaken aiming to inform teenage girls about the adverse outcomes of early marriage and early motherhood through formal and informal education. Parents should be advised to avail of the opportunity of female stipend programme in Bangladesh for their girls to be more educated. Findings of the study suggest that education alone cannot reduce child marriage in Bangladesh as other socioeconomic factors have causal relationship with very young age at marriage. To reduce child marriage and teenage motherhood, the marriage act may be reviewed, and the legal age at marriage may be set at 19 years for females as the country has been passing through a transition period in its economic development. Further, door-step delivery of services for modern contraceptive methods may also reduce teenage motherhood. Healthcare facilities should be made available among the poor and in remote areas for better health of both mother and child in Bangladesh.
